# A PCR method for *VKORC1* G-1639A and *CYP2C9* A1075C genotyping useful to warfarin therapy among Japanese

**DOI:** 10.1186/2193-1801-3-499

**Published:** 2014-09-05

**Authors:** Takashi Tamura, Nobuyuki Katsuda, Nobuyuki Hamajima

**Affiliations:** Department of Epidemiology and Preventive Medicine, Gifu University Graduate School of Medicine, 1-1 Yanagido, Gifu, 501-1194 Japan; Nagoya City Meito Health Center, Nagoya, Japan; Department of Healthcare Administration, Nagoya University Graduate School of Medicine, Nagoya, Japan

**Keywords:** Warfarin, *VKORC1* G-1639A, *CYP2C9* A1075C, SNPs, PCR-CTPP, Genotyping

## Abstract

Warfarin is widely prescribed for patients with the risk of thromboembolism around the world. The inter-individual and inter-racial differences in appropriate dosage depend highly on age, body weight, and genetic factors. A lot of studies including genome-wide association studies revealed that *vitamin K epoxide reductase complex*, *subunit 1* (*VKORC1*) G-1639A and *Cytochrome P450 (CYP) 2C9* A1075C are the most strong genetic factors for determining warfarin effects in Asians and Africans. Since we developed a quick and inexpensive genotyping method, polymerase chain reaction with confronting two-pair primers (PCR-CTPP), the method was applied for these genotypes to examine the possibility to clinical use. Subjects were 436 examinees (117 males and 319 females, aged 32 to 85 years) who attended a health checkup program in Japan. The PCR-CTPP for *VKORC1* G-1639A and *CYP2C9* A1075C was conducted for the subjects, as well as the samples genotyped by DigiTag2 method. The allele frequencies of *VKORC1* G-1639A were 0.085 for *G* and 0.915 for *A*, and those of *CYP2C9* A1075C were 0.979 for *A* and 0.021 for *C*, being in Hardy-Weinberg equilibrium (*p =* 0.658 and *p =* 0.514, respectively). These frequencies were similar to those reported in the HapMap project. Genotyping for both SNPs by PCR-CTPP was replicated by DigiTag2 method. Our results indicated that the PCR-CTPP could be one of the alternative methods for genotyping *VKORC1* G-1639A and *CYP2C9* A1075C for Asians and Africans with similar allele frequencies to Japanese.

## Introduction

Warfarin is one of the most widely prescribed anticoagulants to prevent venous and arterial thromboembolism. This medicine has a very narrow therapeutic range; the high doses increase the risk of bleeding and the low doses do not prevent thromboembolic disorders such as stroke. It is well known that warfarin exhibits large inter-individual and inter-racial differences in the dosage for an appropriate effect. It needs a relatively long time in practice to determine the appropriate dosage for each patient within the optimal prothrombin time measured with an international normalized ratio (PT-INR) range.

Warfarin exerts its anticoagulant effect by inhibiting vitamin K epoxide reductase complex, subunit 1 (VKORC1) (Zimmermann and Matschiner 1974; Suttie 1987). VKORC1 recycles vitamin K 2,3-epoxide to vitamin K hydroquinone, which is essential for glutamyl carboxylation of clotting factors II, VII, IX, and X (Nelsestuen et al. 1974; Stenflo et al. 1974; Suttie 1987). Several studies have reported that the rare mutations in *VKORC1* gene brought clotting factor deficiencies, resulting in warfarin resistance (Li et al. 2004; Rost et al. 2004; D’Andrea et al. 2005). Among them, *VKORC1* G-1639A was identified as one of the most strong causes for warfarin dose requirement (Rieder et al. 2005; Yuan et al. 2005).

*VKORC1* G-1639A is located at the promoter region of *VKORC1* gene (Yuan et al. 2005), and quantitatively changed the expression of VKORC1 protein (Rieder et al. 2005). The promoter with *VKORC1* -1639*G* major allele showed 44% increase in promoter activity compared with the promoter with -1639*A* minor allele (Rieder et al. 2005). These findings have proved that *VKORC1* G-1639A is a single nucleotide polymorphism (SNP) to be one of the most important factors for explaining individual differences in warfarin dosage.

Cytochrome P450 (CYP) is known to play an integral role in biological oxygenation reactions for medicines. CYP2C9 is one of the major isoforms of CYP family, which metabolizes S-warfarin to 7-hydroxywarfarin and 6-hydroxywarfarin (Kaminsky and Zhang 1997). The human *CYP2C9* gene is approximately 55 kb long and located on chromosome 10q24.2 (Meehan et al. 1988; Goldstein and de Morais 1994). Among the genetic variants reported in the human *CYP2C9* gene (Sim and Ingelman-Sundberg 2013), CYP2C9*2 (rs1799853, c.430C > T, p.Arg144Cys) and CYP2C9*3 (rs1057910, c.1075A > C, p.Ile359Leu) alleles are relatively frequent minor alleles in Caucasians, influencing warfarin metabolism (Higashi et al. 2002). CYP2C9*1 allele distributes widely in any ethnic groups, denoted as the wild type (Yin and Miyata 2007). For S-warfarin to 7-hydroxylation, CYP2C9 enzyme activity with *2 allele showed 50% reduction in V_max_ (maximum velocity) and a higher K_m_ (Michaelis constant) compared with the wild type (Rettie et al. 1994; Sullivan-Klose et al. 1996; Yamazaki et al. 1998; Yin and Miyata 2007). CYP2C9 *3 showed a more marked reduction of approximately 90% of the proper clearance (V_max_/K_m_) compared with the wild type (Rettie et al. 1994; Sullivan-Klose et al. 1996; Yamazaki et al. 1998; Yin and Miyata 2007). These reports show that CYP2C9*2 and *3 variants are highly implicated in warfarin metabolism (Aithal et al. 1999). According to the HapMap data, Asians and Africans have no *2 of *CYP2C9*.

In line with these findings, genotyping both *VKORC1* G-1639A and CYP2C9*3 is considered to be useful to find an appropriate individual warfarin dosage for Asians and Africans. In August 2007, the Food and Drug Administration (FDA) in the United States actually approved revisions of attached document for warfarin, following the reports of effects of *VKORC1* and *CYP2C9* on dose requirements (Vladutiu 2008). The FDA recommend that patients possessing these variants be considered to start from a lower initial dose to avoid the risk of bleeding.

For genotyping, several methods have been reported including polymerase chain reaction-restriction fragments length polymorphism (PCR-RFLP) (Erlich et al. 1991), real-time PCR using TaqMan probes (De la Vega et al. 2005), DNA microarray method (Nishida et al. 2007) and quenching probe method (Tani et al. 2009). Each method has limitations, and time and costs for genotyping vary among the methods. We have developed a quick and inexpensive genotyping method named PCR with confronting two-pair primers (PCR-CTPP) (Hamajima et al. 2000; Hamajima 2001), and applied it for many SNPs. This paper describes the feasibility to genotype *VKORC1* G-1639A and CYP2C9*3 with the PCR-CTPP for clinical warfarin use.

### Subjects and methods

#### Subjects

Study subjects were 436 examinees (117 males and 319 females, aged 32 to 85 years) who attended a health checkup program supported by the Nagoya municipal government in 2000. The examinees were inhabitants of Nishi ward at Nagoya city in Japan. A written informed consent to anonymous uses of the residual blood for genetic tests as well as information on demographic characteristics was obtained when the health checkup. About 2 ml of blood was left after a routine test for health check.

Among 489 examinees invited to the study, 468 (95.7%) agreed to provide their residual blood for genetic tests and related information. Three residual blood samples were not available for DNA extraction. In addition, 29 extracted buffy coat samples were used up for other genotyping in previous studies (Hamajima et al. 2001; 2002; 2003). The remaining 436 examinees were subjects in this study.

For both *VKORC1* G-1639A and *CYP2C9* A1075C, 436 examinees were tested by DigiTag2 method to confirm results of genotyping by PCR-CTPP. For *CYP2C9* A1075C, each of two subjects with *1*1, *1*3, and *3*3 among 5,017 participants (3,413 males and 1,604 females, aged 35 to 69 years) in Shizuoka area of the Japan Multi-Institutional Collaborative Cohort Study (J-MICC Study) (Asai et al. 2009) were genotyped by PCR-CTPP in order to confirm whether the genotype information are accorded with those obtained by DigiTag2 method.

#### Genotyping

**Figure 1:**
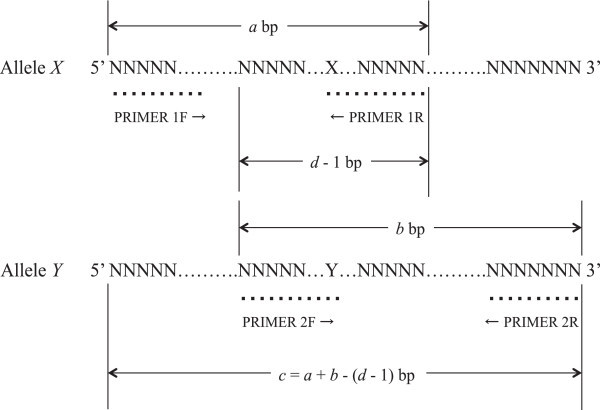
**Logic of PCR-CTPP.** **a**, size for *X* allele; **b**, size for *Y* allele; **c** = *a* + *b* - (*d* - 1), size between primers 1 F and 2R; **d**, Sum of the size of primers 2 F and 1R. PCR-CTPP = Polymerase chain reaction with confronting two-pair primers.

DNA for 436 health check examinees in this study was extracted from the buffy coat fraction preserved at -40°C by a QIAamp DNA Blood Mini Kit (QIAGEN Inc., Valencia, CA). The SNPs were genotyped by PCR-CTPP, and the basic logic has been reported previously (Hamajima et al. 2000; Hamajima 2001). This method requires four primers (two pairs) for each allele specific amplification; F1 and R1 for *X* allele, and F2 and R2 for the *Y* allele (shown in Figure 1). The end base of R1 and F2 should be the position of SNP. The PCR amplifies three different-sized bands of DNA; between F1 and R1, between F2 and R2, and between F1 and R2. The primer sequences for *VKORC1* G-1639A and *CYP2C9* A1075C were shown with the melting temperatures estimated by base sequence algorithm (Breslauer et al. 1986) in Table 1.

**Table 1:** **Primer sequences of PCR-CTPP for** ***VKORC1*** **G-1639A and** ***CYP2C9*** **A1075C**

Primers	Sequences	Tm (°C)
*VKORC1* G-1639A F1	5′ CAC AGA CGC CAG AGG AAG AGA G	64.0
*VKORC1* G-1639A R1	5′ CGT GAG CCA CCG CAC C**T**	65.1
*VKORC1* G-1639A F2	5′ GAA GAC CTG AAA AAC AAC CAT TGG CC**G**	64.4
*VKORC1* G-1639A R2	5′ CTC AGC CTC CCA AGT AGT TTG G	62.1
*CYP2C9* A1075C F1	5′ CCA GGA AGA GAT TGA ACG TGT GAT TG	61.6
*CYP2C9* A1075C R1	5′ TGG TGG GGA GAA GGT CAA **T**	61.3
*CYP2C9* A1075C F2	5′ GCA CGA GGT CCA GAG ATA C**C**	61.7
*CYP2C9* A1075C R2	5′ GAG TTA TGC ACT TCT CTC ACC CG	61.1

*CYP* = *Cytochrome P450*, PCR-CTPP = Polymerase chain reaction with confronting two-pair primers, Tm = Melting temperature, *VKORC1* = *Vitamin K epoxide reductase complex, subunit 1*.

Polymorphic bases are indicated in bold type.

The PCR was performed with initial denaturation at 95°C for 10 min, followed by 30 cycles of denaturation at 95°C for 1 min, annealing at 62°C for *CYP2C9* A1075C and at 61°C for *VKORC1* G-1639A for 1 min, and extension at 72°C for 1 min. The final extension was at 72°C for 5 min.

On the other hand, DNA for participants in Shizuoka area of the J-MICC Study was extracted from the buffy coat fraction by BioRobot® M48 (QIAGEN group, Tokyo, Japan). They were genotyped by DigiTag2 method (NGK INSULATORS, Ltd., Nagoya, Japan) (Nishida et al. 2007).

## Results

**Figure 2:**
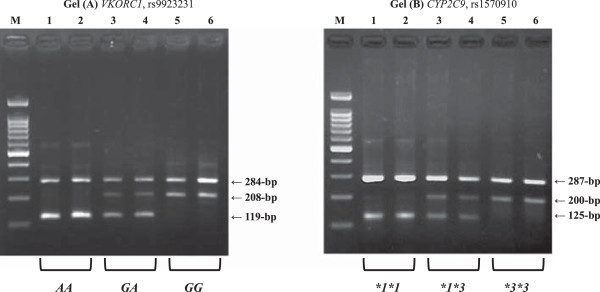
**Representative gels. (A)** *VKORC1* rs9923231 polymorphism. Lane M, a 100-bp ladder; lanes 1 and 2, an *AA* homozygote with fragments of 119-bp and 284-bp; lanes 3 and 4, a *GA* heterozygote with fragments of 119-bp, 208-bp and 284-bp; and lanes 5 and 6, a *GG* with fragments of 208-bp and 284-bp. **(B)** *CYP2C9* rs1570910 polymorphism. Lane M, a 100-bp ladder; lanes 1 and 2, a *1*1 homozygote with fragments of 125-bp and 287-bp; lanes 3 and 4, a *1*3 heterozygote with fragments of 125-bp, 200-bp and 287-bp; and lanes 5 and 6, a *3*3 with fragments of 200-bp and 287-bp. *CYP* = *Cytochrome P450*, *VKORC1* = *Vitamin K epoxide reductase complex, subunit 1*.

Figure 2 shows actual gels for *VKORC1* G-1639A (rs9923231) in Gel (A) and *CYP2C9* A1075C (rs1570910) in Gel (B) obtained by PCR-CTPP. Each Sample (from one to six in Figure 2) was different one. Among 436 subjects, there was no one with *3*3 of *CYP2C9* because of the less frequency. We therefore selected the subjects with *3*3 among participants of Shizuoka area in the J-MICC Study in order to genotyping by PCR-CTPP.

The genotype information (*1*1, *1*3, and *3*3: each of two samples) of Shizuoka area obtained by DigiTag2 method was accorded with that obtained by PCR-CTPP. There was no genotype information for *VKORC1* G-1639A in Shizuoka area of the J-MICC Study. Both *VKORC1* G-1639A and *CYP2C9* A1075C for 436 examinees were replicated by DigiTag2 method.

Table 2 shows the genotype frequencies of *VKORC1* G-1639A and *CYP2C9* A1075C. Each allele frequency was all in Hardy-Weinberg equilibrium. As failed genotyping, there were 10 (2.3%, n = 436) for *VKORC1* G-1639A, and four (0.9%, n = 436) for *CYP2C9* A1075C. Comparisons between these allele frequencies and those reported in the other study and the HapMap project were shown in Table 3, which were all fairly similar.

**Table 2:** **Genotypes and allele frequencies for** ***VKORC1*** **G-1639A and** ***CYP2C9*** **A1075C obtained by PCR-CTPP**

Gene	N	Frequency (%)	Allele frequency		Hardy-Weinberg equilibrium ***p***
*VKORC1* (rs9923231, c.-1639G > A)					
Nagoya					
*GG*	2	0.5	*G*	0.085	0.514
*GA*	68	16.0	*A*	0.915	
*AA*	356	83.5			
Total	^a^426	100			
*CYP2C9* (rs1057910, c.1075A > C)					
Nagoya					
*AA* (*1*1)	414	95.8	*1	0.979	0.658
*AC* (*1*3)	18	4.2	*3	0.021	
*CC* (*3*3)	0	0			
Total	^b^432	100			

*CYP* = *Cytochrome P450*, PCR-CTPP = Polymerase chain reaction with confronting two-pair primers, *VKORC1* = *Vitamin K epoxide reductase complex, subunit 1*.

^a^Data was missing for 10 subjects with unsuccessful genotyping.

^b^Four were failed.

**Table 3:** **Comparisons of allele frequencies for** ***VKORC1*** **G-1639A and** ***CYP2C9*** **A1075C**

Allele frequency	***VKORC1*** c.-1639G > A (rs9923231)		***CYP2C9*** c.1075A > C (rs1057910)	
	***G***	***A***	***A***	***C***
Nagoya citizen	0.085	0.915	0.979	0.021
HapMap projects				
African	0.978	0.022	1.000	0.000
Caucasian	0.602	0.398	0.942	0.058
Chinese	0.058	0.942	0.953	0.047
Japanese	0.099	0.901	0.977	0.023
Japanese in Shizuoka area				
Yoshizawa, et al. 2009	0.082	0.918	0.979	0.021

*CYP* = *Cytochrome P450*, *VKORC1* = *Vitamin K epoxide reductase complex, subunit 1*.

## Discussion

In this study, we finely conducted the genotyping for *VKORC1* G-1639A and *CYP2C9* A1075C using PCR-CTPP method. The allele frequencies were confirmed to be reasonable by the comparisons with those reported in the HapMap data. Both SNPs for 436 examinees were replicated by DigiTag2 method. These results have proved that PCR-CTPP is applicable for genotyping *VKORC1* G-1639A and *CYP2C9* A1075C.

For genotyping SNPs, several studies have recommended their options (Erlich et al. 1991; De la Vega et al. 2005; Nishida et al. 2007; Tani et al. 2009). Among them, PCR-RFLP and TaqMan method are commonly used. Nonetheless, they were not necessarily confirmed to have the feasibility or be reasonable for genotyping *VKORC1* G-1639A and CYP2C9 *3 for tailored warfarin use. Besides, former needs a longer time and later is quick with expensiveness for genotyping.

The main difference between PCR-CTPP and PCR-RFLP is that PCR-CTPP dose not need incubation with restriction enzymes for PCR product digestion. PCR-CTPP therefore has an advantage of lowest cost only for primers and polymerase. There is no restriction enzyme step in PCR-CTPP, which means that we could conduct the genotyping only half of the time compared with PCR-RFLP. Recently, use of real-time PCR was also developed for genotyping SNPs (De la Vega et al. 2005), although the cost per one sample is still too expensive (about two dollars), and the method needs exclusive machines in laboratory as in DigTag2 and Q-probe methods (Nishida et al. 2007; Tani et al. 2009). In contrast, the cost of PCR-CTPP is only half dollar per one sample (as costs of primers and Taq polymerase).

Some technical problems have been reported for PCR-CTPP (Hamajima et al. 2002). The strength of bands is dependent on the balance in melting temperature of each primer. Addition of one base to a primer changes its melting temperature and causes distraction of the balance of the bands strength. The melting temperatures varied in a wide range (shown in Table 1). The optimum primers and all the condition in this study were determined after several unsuccessful combinations. However, a further option that one primer amplification of PCR-CTPP products (OPA-CTPP) has been developed for resolving these problems (Yin et al. 2012).

As limitations, we could not verify the detection for *2 of *CYP2C9* because almost all Japanese have no *2 of *CYP2C9* as well as other Asians and Africans*.* It needs to confirm those with the *2 using PCR-CTPP for Caucasian. Additionally, it may also need to confirm those with *5, *6, *8, and *11 in relation to warfarin dose for Africans, when the frequencies are considered to be high on the clinical practice (Limdi et al. 2008; Scott et al. 2009).

The proportions of failed genotyping seem to be relatively high. These phenomena, however, would be explained by buffy condition (i.e., lowered concentration, insufficient amount of sample, or DNA degradation over time).

Using PCR-CTPP, we have already succeeded in other tailored therapy and prevention. The details were described elsewhere (Tamura et al. 2011, 2012). Tailored warfarin therapy in clinical practice could also be supported by PCR-CTPP, especially among Japanese.

## Conclusion

We suggested that PCR-CTPP would be useful when genotyping *VKORC1* G-1639A and CYP2C9 *3 is needed in tailored warfarin use for Asians and Africans. Application, experience, and further data in real patients with warfarin therapy would be warranted hereafter. Establishment in other tailored therapy is also expected.

## Abbreviations

CYP: Cytochrome P450
FDA: Food and drug administration
J-MICC Study: Japan Multi-Institutional Collaborative Cohort Study
OPA-CTPP: One primer amplification of polymerase chain reaction with confronting two-pair primers products
PCR-CTPP: Polymerase chain reaction with confronting two-pair primers
PCR-RFLP: Polymerase chain reaction-restriction fragments length polymorphism
PT-INR: Prothrombin time measured with an international normalized ratio
SNP: Single nucleotide polymorphism
VKORC1: Vitamin K epoxide reductase complex, subunit 1.
